# Gute Therapieverträglichkeit und Lebensqualität nach primär organerhaltender Therapie des frühen Rektumkarzinoms: Ergebnisse der TREC-Studie

**DOI:** 10.1007/s00066-023-02079-5

**Published:** 2023-04-21

**Authors:** Georg W. Wurschi, Andrea Wittig

**Affiliations:** 1 Klinik für Strahlentherapie und Radioonkologie, Universitätsklinikum Jena, Jena, Deutschland; 2 Clinician Scientist Programm, Interdisziplinäres Zentrum für Klinische Forschung, Universitätsklinikum Jena, Jena, Deutschland

## Hintergrund

Beim lokal fortgeschrittenen Rektumkarzinom sind verschiedene Konzepte mit intendiertem Organerhalt im Rahmen klinischer Studien erfolgreich geprüft worden. Die vielversprechenden Komplettremissionsraten und die gute Verträglichkeit lassen dieses Vorgehen in Deutschland zunehmend zum Standard werden. Bei frühen Tumorstadien (Union internationale contre le cancer, UICC, Stadium I bzw. T1/T2-N0-Tumoren) ist bisher die primäre Resektion hingegen Therapie der Wahl, birgt aber gerade bei multimorbiden alten Patienten ein höheres Op.-assoziiertes Nebenwirkungsrisiko. Für diese Patientengruppe sind bisher nur wenige Arbeiten verfügbar und insbesondere der Organerhalt wird im UICC-Stadium I dabei kaum betrachtet.

Die britische TREC-Studie untersuchte ein organerhaltendes Vorgehen beim Adenokarzinom des Rektums im UICC-Stadium I. Eine Kurzzeitbestrahlung (5 × 5 Gy) mit anschließender transanaler endoskopischer Resektion wurde randomisiert mit einer totalen mesorektalen Exzision (TME) verglichen [2]. In der jetzt publizierten Unteranalyse der Studie wird auf die Verträglichkeit und das onkologische Outcome im 3‑Jahres-Follow-up bei älteren Patienten fokussiert. In dieser Arbeit wurde die Subgruppe der Patienten mit organerhaltender Therapie ausgewertet, zudem weitere Patienten aus einem sekundären Register, die aufgrund mangelnder Operabilität nicht randomisiert werden konnten [4].

## Patienten und Methodik

In der Studie wurden 61 Patienten aufgrund von Komorbiditäten, Gebrechlichkeit oder Alter als nicht operabel eingestuft und folglich nicht randomisiert. Diese wurden in ein sekundäres Register eingeschlossen und nach organerhaltender Therapie mit standardisierten Fragebögen zur Lebensqualität und (kolorektalen) Funktionalität (EORTC-QLQ C30 & CR29, COREFO, EQ-5D-3L) für 36 Monate nachverfolgt. Die Patienten in dem Register wurden im Rahmen der Studie mit zwei weiteren Patientengruppen verglichen: mit (a) 27/123 Patienten, die in der TREC-Studie dem Arm zugeordnet wurden, der das organerhaltende Vorgehen beinhaltete, und (b) einer gleichaltrigen Referenzkohorte der britischen Bevölkerung.

## Ergebnisse

Patienten in der Registerstudie waren älter als diejenigen in der randomisierten Studie (mittleres Alter 74 Jahre, Interquartilabstand [IQR] 67–80 Jahre). Bei den nicht randomisierten Patienten war nach 3 Jahren ein Organerhalt in 92 % der Fälle möglich bei einer rezidivfreien Überlebensrate der Kohorte von 91 %. Innerhalb der 3 Jahre waren 9 Patienten verstorben, davon 5 an nicht karzinomassoziierten Ursachen. Patienten in der Registerstudie schnitten prätherapeutisch (Baseline) und in der Nachsorge hinsichtlich der Lebensqualität und anderer sozial/physisch bzw. funktionell relevanter Scores schlechter ab als die Patienten, bei denen die Randomisation erfolgte.

Vor Therapiebeginn hatten die Registerpatienten einen höheren Score als der alterskorrigierte Durchschnitt der britischen Bevölkerung ohne Tumordiagnose. Patienten in der Registerstudie hatten keine klinisch relevant schlechtere Lebensqualität oder Funktionswerte als Patienten in der randomisierten Studie (> 10 Punkte). In sozialen und funktionellen Items waren in beiden Gruppen zwischenzeitlich Beeinträchtigungen nachweisbar, die sich jedoch binnen 6–12 Monaten normalisierten. Hinsichtlich Stuhlinkontinenz bzw. -unregelmäßigkeiten waren nach 36 Monaten noch leichte Beeinträchtigungen im Vergleich zur Allgemeinbevölkerung auszumachen. Hinsichtlich sexueller Funktion scheint ebenfalls eine geringe Beeinträchtigung vorzuliegen, allerdings lagen diese Angaben nur von einem geringen Teil der Patienten vor.

## Schlussfolgerungen der Autoren

Die Autoren finden eine gute Verträglichkeit des Konzepts der hypofraktionierten Strahlentherapie mit transanaler endoskopischer Resektion bei hohen Raten hinsichtlich Organerhalt und gutem Funktionsstatus. Sie empfehlen diese Therapiealternative insbesondere für Patienten mit hohem Op.-Risiko.

## Kommentar

Unseres Erachtens wird mit dieser Analyse ein vielversprechender Ansatz zur risikoadaptierten Therapie des Stadium-I-Rektumkarzinoms bei älteren Patienten vorgestellt. Neben dem onkologischen Outcome müssen in dieser, in Zukunft immer größer werdenden, Patientengruppe Risikobewertung und Durchführbarkeit der Therapie vor dem Hintergrund von Komorbiditäten und Gebrechlichkeit gewichtet werden. Kurze Krankenhausaufenthalte und der Verzicht auf invasivere Prozeduren sind dabei ein Schlüssel zum Erfolg und werden in diesem Protokoll aufgegriffen.

In dem relativ kleinen Kollektiv zeigten sich gleichwertige Ergebnisse hinsichtlich der Rezidivrate (10 % im 3‑Jahres-Follow-up) verglichen mit Ergebnissen nach TME oder endoskopischer Resektion nach hypofraktionierter Strahlentherapie im Stadium I [5, 9, 10].

Das Vorgehen der britischen Studiengruppe fokussiert sich auf frühe Tumorstadien und steht damit im Kontrast zu den Studien zum intendierten Organerhalt beim lokal fortgeschrittenen Rektumkarzinom (UICC-Stadien II/III), wie bspw. OPRA [3] oder der aktuellen CAO/ARO/AIO-16 Studie. Bei diesen wird die notwendige Therapieeskalation zur Sicherstellung einer längerfristigen Tumorkontrolle mittels umfangreicher Systemtherapie erreicht, die bei älteren Patientenkollektiven aufgrund internistischer Komorbiditäten und eingeschränkter Verträglichkeit möglicherweise schwer realisierbar ist.

Eine klinisch praktikable Stratifizierung von „Gebrechlichkeit“ ist für die Identifizierung von Patienten mit geringer funktioneller Reserve, die nicht von einer Operation profitieren, entscheidend [1, 8]. Hierfür existieren neben dem komplexen „Comprehensive Geriatric Assessment“ (CGA) kompaktere geriatrische Testmodule, die auch von den europäischen und amerikanischen chirurgischen bzw. geriatrischen Fachgruppen beim Rektumkarzinom empfohlen werden [6] und Gegenstand aktueller klinischer Prüfungen sind [7].

Im Stadium I ist in Deutschland die primäre Resektion nach wie vor Leitlinienstandard. Es bedarf zusätzlicher Evidenz, um das insbesondere funktionell vorteilhafte Konzept durchzusetzen. Einen Beitrag hierzu wird die aktuell laufende Folgestudie „STAR TREC“ (NCT02945566) liefern.

## Fazit

Die Autoren um Gilbert et al. fokussieren in dieser Subgruppenanalyse der TREC-Studie auf die Gruppe älterer und gebrechlicherer Patienten, für die durch eine hypofraktionierte Bestrahlung mit organerhaltender Resektion im Vergleich zur TME eine überzeugend gute Verträglichkeit bei vergleichbarem onkologischem Ergebnis erreicht wird.

*Georg W. Wurschi und Andrea Wittig, Jena*
